# Telehealth for patients at high risk of cardiovascular disease: pragmatic randomised controlled trial

**DOI:** 10.1136/bmj.i2647

**Published:** 2016-06-01

**Authors:** Chris Salisbury, Alicia O’Cathain, Clare Thomas, Louisa Edwards, Daisy Gaunt, Padraig Dixon, Sandra Hollinghurst, Jon Nicholl, Shirley Large, Lucy Yardley, Tom Fahey, Alexis Foster, Katy Garner, Kimberley Horspool, Mei-See Man, Anne Rogers, Catherine Pope, Alan A Montgomery

**Affiliations:** 1 Centre for Academic Primary Care, School of Social and Community Medicine, University of Bristol, Bristol BS8 2PS, UK; 2 School of Health and Related Research (ScHARR), University of Sheffield, Sheffield, UK; 3 Bristol Randomised Trials Collaboration, School of Social and Community Medicine, University of Bristol, Bristol, UK; 4 NHS England South (Wessex), Southampton, UK; 5 Department of Psychology, University of Southampton, Southampton, UK; 6 Department of General Practice, Royal College of Surgeons in Ireland, Dublin, Republic of Ireland; 7 Faculty of Health Sciences, University of Southampton, Southampton, UK; 8 Nottingham Clinical Trials Unit, Nottingham Health Science Partners, Queen’s Medical Centre, Nottingham, UK

## Abstract

**Objective** To assess whether non-clinical staff can effectively manage people at high risk of cardiovascular disease using digital health technologies.

**Design** Pragmatic, multicentre, randomised controlled trial.

**Setting** 42 general practices in three areas of England.

**Participants** Between 3 December 2012 and 23 July 2013 we recruited 641 adults aged 40 to 74 years with a 10 year cardiovascular disease risk of 20% or more, no previous cardiovascular event, at least one modifiable risk factor (systolic blood pressure ≥140 mm Hg, body mass index ≥30, current smoker), and access to a telephone, the internet, and email. Participants were individually allocated to intervention (n=325) or control (n=316) groups using automated randomisation stratified by site, minimised by practice and baseline risk score.

**Interventions** Intervention was the Healthlines service (alongside usual care), comprising regular telephone calls from trained lay health advisors following scripts generated by interactive software. Advisors facilitated self management by supporting participants to use online resources to reduce risk factors, and sought to optimise drug use, improve treatment adherence, and encourage healthier lifestyles. The control group comprised usual care alone.

**Main outcome measures** The primary outcome was the proportion of participants responding to treatment, defined as maintaining or reducing their cardiovascular risk after 12 months. Outcomes were collected six and 12 months after randomisation and analysed masked. Participants were not masked.

**Results** 50% (148/295) of participants in the intervention group responded to treatment compared with 43% (124/291) in the control group (adjusted odds ratio 1.3, 95% confidence interval 1.0 to 1.9; number needed to treat=13); a difference possibly due to chance (P=0.08). The intervention was associated with reductions in blood pressure (difference in mean systolic −2.7 mm Hg (95% confidence interval −4.7 to −0.6 mm Hg), mean diastolic −2.8 (−4.0 to −1.6 mm Hg); weight −1.0 kg (−1.8 to −0.3 kg), and body mass index −0.4 ( −0.6 to −0.1) but not cholesterol −0.1 (−0.2 to 0.0), smoking status (adjusted odds ratio 0.4, 0.2 to 1.0), or overall cardiovascular risk as a continuous measure (−0.4, −1.2 to 0.3)). The intervention was associated with improvements in diet, physical activity, drug adherence, and satisfaction with access to care, treatment received, and care coordination. One serious related adverse event occurred, when a participant was admitted to hospital with low blood pressure.

**Conclusions** This evidence based telehealth approach was associated with small clinical benefits for a minority of people with high cardiovascular risk, and there was no overall improvement in average risk. The Healthlines service was, however, associated with improvements in some risk behaviours, and in perceptions of support and access to care.

**Trial registration** Current Controlled Trials ISRCTN 27508731.

## Introduction

The growing prevalence of long term conditions means that new and more efficient approaches to healthcare delivery are needed that support people to manage their own care, with less reliance on consultations with expensively trained healthcare professionals. Effective self management, as part of a shift in the management of long term conditions, can help improve health outcomes and reduce costs.^1^ ^2^ Many countries are exploring a greater use of technologies, such as the internet, remote monitoring, and telephone support as a way of expanding provision and increasing access to care for a large number of people at relatively low cost. In the United Kingdom, current policy envisages these “telehealth” approaches as having potential to transform the delivery of healthcare to make the national health service sustainable for the future.^3^ In the United States, the Veterans Health Administration has enrolled more than 50 000 people in a home telehealth programme,^4^ ^5^ and in Europe the Renewing Health Consortium is evaluating telehealth programmes in nine countries.^6^

**Figure 1:**
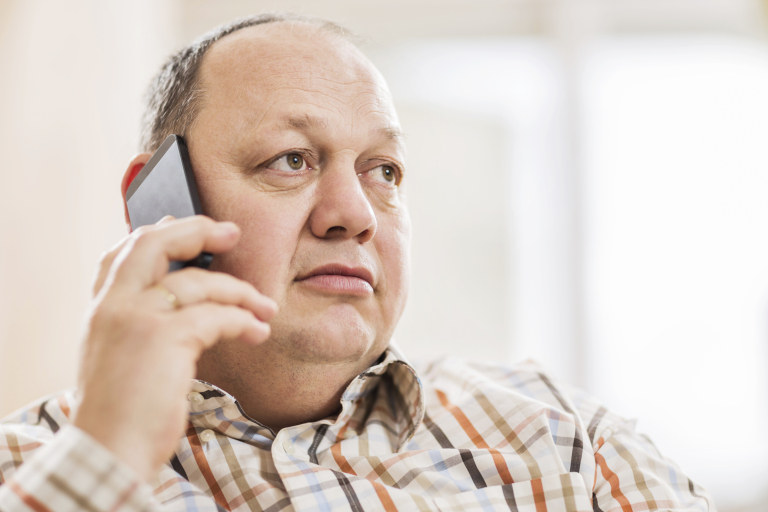


The volume of literature on the effectiveness of specific telehealth interventions is burgeoning, with promising effects for some applications. However, recent reviews have highlighted that much of the evidence is of poor quality; results are inconsistent; there is a lack of theoretical underpinning, which makes it difficult to interpret the mixed results; and there is some evidence of publication bias in favour of positive results.^7^ ^8^ ^9^ ^10^ Furthermore, focusing on specific applications or technologies in isolation is of limited value since they need to be considered in the context of their implementation within the healthcare system. In practice, large scale healthcare programmes based on telehealth involve the combined use of technologies—for example, online programmes or remote monitoring with telephone support from advisors following computerised algorithms. In the recent five year strategic plan for the NHS, it is argued that evaluation is needed of “combinatorial innovation,” in which a range of technologies are provided in combination with new ways of working.^11^ ^12^ Few rigorous pragmatic studies have been done on implementation of this approach in the real world.^8^ Furthermore, a key aspect of the argument for telehealth is increased efficiency, but there are few studies incorporating economic analyses, and the limited evidence available suggests that many telehealth interventions are not cost effective.^13^

We conducted a research programme to develop a conceptual model for the effective use of telehealth in long term conditions, based on literature reviews,^14^ ^15^ qualitative research,^16^ and surveys of patients’ views.^17^ Designated the telehealth in chronic disease (TECH) model, this builds on existing approaches such as the chronic care model by creating a framework for improving the management of chronic diseases through telehealth.^18^ We used this model to design the Healthlines service for the management of long term conditions, based on the combined use of internet based health applications that had evidence of effectiveness supported by non-clinically qualified staff working using tailored computerised algorithms.^19^

We evaluated the Healthlines service through linked pragmatic multicentre randomised controlled trials with nested process and economic evaluations in two exemplar conditions: depression and increased cardiovascular risk. This paper reports the findings for patients with an increased cardiovascular risk. Although hypertension, obesity, and hyperlipidaemia are often considered as long term conditions, it is more appropriate to consider them as risk factors, with their combined effect determining overall cardiovascular risk.^20^ This was considered an appropriate exemplar because of the high number of people affected (10% of adults aged 35-74 in England and Wales have a 10 year cardiovascular risk ≥20%),^21^ which has serious health consequences as a result of heart attacks, strokes, kidney disease, and other problems. Cardiovascular disease causes 28% of deaths in England, accounts for 10% of all hospital admissions, and involves an annual expenditure in England of almost £7bn.^22^ A low cost intervention that could be made widely available to large numbers of people could have a beneficial impact at a population level even if the effect for an individual was small.

Evidence exists for the effectiveness of specific relevant technological approaches, such as home blood pressure monitoring,^23^ mobile phone applications to support smoking cessation,^24^ and online interventions for weight loss.^25^ This evidence provided a good basis for the hypothesis that combining these “active ingredients” and implementing them within a new telehealth model of care would be effective and cost effective. Furthermore, the introduction in 2008 of the NHS Health Check programme was likely to identify a large number of people at high cardiovascular risk, and there was a need to explore ways to expand provision of care to manage them once they had been identified.^26^

Our hypothesis was that the Healthlines service for patients with high cardiovascular risk would be more clinically effective and cost effective than usual care, while also improving participant’s quality of life, risk behaviours, and experience of care.

## Methods

### Design

This was a pragmatic, multicentre, randomised controlled trial comparing the Healthlines service in addition to usual care versus usual care alone in adults with a high risk of cardiovascular disease. The study was registered before recruitment of the first participant, and the study protocol has been published.^19^ After the trial commenced there were no important changes to the methods, apart from the addition of a nested substudy of different forms of information in the patient invitation to assess the impact on participant recruitment rates. This did not alter the design or outcomes for the main trial; results of this substudy are published elsewhere.^27^

### Participants

Patients eligible for the trial were aged between 40 and 74 years, had a risk of a cardiovascular event in the next 10 years of 20% or more calculated using the QRISK2 score,^21^ and had one or more of the following modifiable risk factors (systolic blood pressure ≥140 mm Hg, body mass index ≥30, being a current smoker, or any combination of these). Participants required access to a telephone, the internet, and an email address. We excluded people who had a previous cardiovascular event; were pregnant or planning pregnancy; had a serious mental health problem, dementia, severe learning disability, or substance dependency; were receiving palliative care; or were unable to communicate verbally in English.

Participants were recruited from 42 general practices covering populations with a range of sociodemographic characteristics in and around Bristol, Sheffield, and Southampton, England. We used patients’ medical records to identify those who had at least one modifiable risk factor and estimated 10 year cardiovascular risk of 18% or more (we were over-inclusive at the initial screening stage because QRISK2 scores based on historical records may not reflect current risk and we wanted to invite potentially eligible people to have an updated risk assessment). A random sample of these potentially eligible patients in each practice was sent information about the study by post, after general practitioners screened the list for patients with known exclusion criteria. We sent information to between 250 and 285 patients in each practice, altering the sampling fraction over time to achieve our recruitment targets. A researcher telephoned patients who expressed an interest in the study to conduct initial eligibility screening and then invited them for an assessment of cardiovascular risk status by a practice nurse at their participating general practice. The nurse measured the patients’ blood pressure, weight and height, smoking status, and total cholesterol to high density lipoprotein cholesterol ratio, and collected all other relevant information needed to calculate the patient’s QRISK2 score (see supplementary appendix 1). Patients who had a QRISK2 score of 20% or more and had one or more of the specified modifiable risk factors completed a baseline questionnaire and consent form, either online or by post.

### Intervention and control

Participants in the control group could continue to receive all care normally provided by the NHS, but had no contact with the Healthlines service. Usual care involved management of cardiovascular risk factors by primary care clinicians, including, in some cases, referral to community services for advice about smoking cessation and weight management.

Participants in the intervention arm received support from the Healthlines service in addition to usual NHS care. The Healthlines service is a multifaceted intervention, incorporating a range of strategies to address the various components of the TECH model (see box 1).^18^ The intervention is based around regular telephone calls from a health advisor, supported by patient specific tailored algorithms and standardised scripts generated through a computerised behavioural management programme. This programme was originally developed and successfully evaluated in the United States by Bosworth et al and includes a series of modules on subjects such as drug adherence, diet, and smoking cessation.^28^ ^29^ The standardised scripts generated by the software were based on recognised principles for behaviour change, such as stimulus control, problem solving, cognitive restructuring, and goal setting.^30^ We modified the programme to reflect English management guidelines and referral options, wrote additional modules with new content, and adapted the language to suit an English population.

**Box 1: Components of the Healthlines cardiovascular disease risk intervention, reflecting the TECH conceptual model, with examples of strategies**

Computerised behaviour management programme, providing interactive scripts used by health advisors
  Modules include:
    o Knowledge about cardiovascular risk and healthy lifestyles
    o Review of drugs and side effects
    o Optimisation of drugs for blood pressure lowering
    o Home blood pressure monitoring
    o Review of statins
    o Support for drug adherence
    o Smoking and nicotine replacement therapy
    o Healthy eating
    o Weight loss and Orlistat
    o Alcohol use
    o Exercise
Motivational interviewing. All health advisors were trained in motivational interviewing
Self monitoring and feedback—for example, blood pressure online self monitoring programme with automated feedback
Treatment optimisation and intensification—health advisors monitor treatment response, and send emails to clinicians to intensify treatment when necessary, along with reminders of treatment guidelines
Addressing drug adherence—monthly review, scripts incorporated evidence based strategies to promote adherence
Improving care coordination—sharing all information sent to clinicians with patients
Supporting primary care—all aspects of the intervention designed to support rather than duplicate primary care
Strategies to promote engagement of patients—through continuity of care with the same advisor; providing technical support with getting online
Strategies to promote engagement of primary care clinicians—emphasising the evidence based nature of intervention components and how it can support their work in primary care

For the initial assessment, health advisors contacted each participant by telephone to discuss their health needs and to agree on specific goals. After the initial call, the advisors telephoned each participant approximately every month for one year. The software was interactive and provided different computerised scripts so that the content of each call was tailored to meet each participant’s particular needs and goals. The software provided health advisors with links to relevant and quality assured online resources and applications to support self management (for example, to help with losing weight or stopping smoking), and the advisors sent these links to participants by email or post. To avoid an anonymous “call centre” approach, the same advisor telephoned each participant on each occasion when possible, since our earlier qualitative research had identified a relationship with the advisor as an important factor in engaging prospective participants.^15^ The Healthlines service was designed to improve access to care and was available until 8 pm on weekdays and 2 pm on Saturdays.

Participants were also provided with access to a Healthlines web portal where they could obtain further information about cardiovascular disease, access other online resources, request a call-back from Healthlines staff, see copies of letters to their general practitioner, and use a blood pressure self monitoring system. Participants with a baseline systolic blood pressure of 140 mm Hg or more were offered a validated home blood pressure monitor (Omron, M3) by their practice nurse, requested to take their blood pressure twice daily for the first week and weekly thereafter, and to upload their readings to the Healthlines portal. The portal calculated average readings over the previous six days initially and thereafter over the previous six weeks. Using these readings, participants were automatically advised by the portal whether their blood pressure was within their target, when to take their blood pressure again, and what to do if their blood pressure was too high or too low. Target blood pressure was based on UK guidelines,^31^ although an individual’s target could be modified by his or her general practitioner. At each telephone contact, health advisors reviewed average blood pressure readings, and participants with above target readings were asked to see their doctor to review their treatment. Advisors sent an email to the general practice, attaching details of the patient’s recent blood pressure readings and a summary of guidelines from the National Institute for Health and Care Excellence about recommended steps for intensifying treatment.

The Healthlines advisors were not clinically qualified but had experience of working as health advisors for NHS Direct and had a further three weeks of training in health coaching, motivational interviewing, treatment options (including drugs) for hypertension, smoking and obesity, and use of the Healthlines computerised management programme.

The Healthlines service was originally hosted by NHS Direct, which provided a range of telehealth services through a network of call centres and a nationally recognised website. When NHS Direct closed in March 2014, delivery of the intervention was paused for two months while the staff and computer systems were transferred to a new provider (Solent NHS Trust). Although the Healthlines service resumed unaltered after this hiatus, about two thirds of participants experienced some disruption, and some participants could not receive the full number of telephone calls during their 12 month follow-up period.

### Outcomes

The primary outcome was the proportion of participants responding positively to treatment, defined as maintaining or reducing their 10 year cardiovascular risk 12 months after randomisation, estimated using the QRISK2 score. Since cardiovascular risk increases with age, maintaining or reducing risk over 12 months requires an improvement in at least one modifiable risk factor. We treated the QRISK2 score (continuous) as a secondary outcome. The estimate of risk was based on data collected at an assessment by a nurse or healthcare assistant at the participant’s general practice at six and 12 months after recruitment using the same procedures as used at baseline (see supplementary appendix 1). We calculated follow-up QRISK2 scores by updating age and values for modifiable risk factors only. Other variables such as diagnoses of atrial fibrillation or diabetes were not altered to avoid bias from the greater attention paid to participants in the intervention arm.

Cardiovascular risk is a composite outcome, and the individual risk factors of blood pressure, weight (and body mass index), smoking, and cholesterol level were important secondary outcomes. Other secondary outcomes were quality of life, exercise, diet, satisfaction with treatment received and with amount of support received, perceived access to care, self management skills and self efficacy, drug adherence, health literacy, use of telehealth, and perceptions of care coordination. Table 6 lists the specific measurement instruments used. Secondary outcome measures were collected through patient questionnaires, completed online or by post at baseline and six and 12 months after randomisation. We obtained data about prescriptions and primary care consultations from general practice records and details on use of the intervention from Healthlines records. Potential serious adverse events were identified through reports from participants or health professionals, further inquiry about hospital admissions reported in outcome questionnaires, or admissions, deaths, or other potential serious adverse events identified through review of primary care notes at the end of the trial. We logged all such events with a description of the event and an assessment of expectedness, relatedness, and seriousness and we reported to the trial monitoring committee, sponsor, and ethics committee as appropriate.

### Sample size

The sample size was chosen pragmatically, taking into account the size of effect that would be likely to influence practice and might be feasible to detect in a trial of reasonable size. Based on a previous study we assumed that 35% of participants in the control arm would maintain or reduce their cardiovascular risk over 12 months.^32^ Including 240 participants in each trial arm for analysis would provide 80% power (5% α) and 90% power (1% α) to detect differences of 13 and 18 percentage points, respectively. Assuming 20% attrition, we therefore aimed to recruit 600 participants, 300 in each trial arm.

### Randomisation and masking

Participants who provided consent were randomly allocated in 1:1 ratio to the intervention or usual care group. Allocation was made using a web randomisation system hosted by the Bristol Randomised Controlled Trials Collaboration, and automated to ensure concealment. Randomisation was stratified by location of recruitment (Bristol, Sheffield, or Southampton) and minimised by general practice and baseline QRISK2 score. Researchers notified the participants of their allocation by email. Participants were not masked to treatment allocation. Practice nurses or healthcare assistants collected data for the QRISK2 score and may have been aware of treatment allocation at follow-up, but the variables of relevance on smoking (validated using a carbon monoxide monitor), blood pressure, and cholesterol level were all based on objective quantitative data. All other outcome data were collected by participant self report or electronic download from medical records and were entered and analysed blinded to treatment allocation.

### Statistical analysis

Analysis was conducted according to CONSORT guidelines, following an analysis plan agreed in advance with the independent trial steering committee and data monitoring committee. We used descriptive statistics to compare baseline characteristics of trial participants by allocated arm. The primary analysis of response to treatment after 12 months was conducted using a mixed effects logistic regression model adjusted for site, baseline QRISK2 score, and general practice (as a random effect). Participants were analysed according to allocated arm. We conducted sensitivity analyses of the primary outcome using: the assumption that all participants were exactly one year older at 12 months’ follow-up, simple imputation of missing outcome data that assumed no treatment response, multiple imputation of missing data, exclusion of general practitioner’s practice as a random effect, and adjustment by time between randomisation and follow-up. By fitting interaction terms between trial arm and subgroup variables, we investigated whether any effect of the Healthlines intervention on the primary outcome differed according to subgroups defined by sex, age, baseline QRISK2 score, and presence or absence of each of the modifiable risk factors (hypertension, obesity, smoking) at baseline.

In secondary analysis of the primary outcome, we estimated the complier average causal effect of the Healthlines intervention when received as intended. We described compliance as little or none (two or fewer telephone calls), partial (three to 11 calls), or full (12 or 13 calls). We estimated the complier average causal effect at 12 months using principal stratification in two ways: classifying partial compliers as either non-compliers or full compliers.^33^

Secondary outcomes were analysed in a similar manner to the primary outcome. We estimated between group effects using linear, logistic, or binomial mixed effects regression models, adjusted for stratification and minimisation variables and value of the outcome at baseline. Participants were analysed as randomised without imputation of missing data. To reduce the number of statistical comparisons, we estimated between group differences for secondary outcomes (other than cardiovascular risk factors) only at the final 12 month follow-up time point. We described serious adverse events by study arm.

We assessed the cost effectiveness of the Healthlines intervention from an NHS perspective at 12 months from randomisation. Cost effectiveness was not listed as a secondary outcome in the trial registry because we viewed it as an approach to analysis rather than as an outcome; however, assessment of cost effectiveness was specified a priori as an aim in the registry and described in the published protocol. The methods and results of the economic evaluation will be described in detail elsewhere. In brief, we compared health system costs with incremental quality adjusted life years, measured using the EQ-5D-5L generic quality of life questionnaire^34^ at baseline and six and 12 months post-randomisation, to produce an estimate of net monetary benefit. We also developed a cohort simulation model in order to estimate the cost effectiveness of the intervention over the estimated remaining lifetime of trial participants.

All analyses were conducted using Stata version 13 MP2. The trial was registered prospectively with Current Controlled Trials (ISRCTN 27508731).

### Patient involvement

There was strong and valuable patient and public involvement throughout the Healthlines research programme. Two service user groups (Mental Health Research Network and NHS Direct user group) provided feedback on the initial questionnaire about patients’ preferences and needs in relation to telehealth, which helped to inform the intervention design.^17^ Two representatives of these groups became members of the management group for the five year research programme. They contributed to the design of the patient survey,^17^ participated in a workshop to develop the TECH model that underlies the intervention,^18^ and became members of the trial steering committee for the randomised trial.^19^ They commented on the acceptability of the intervention to potential participants and obtained feedback from their user groups on the outcome measures. At the end of the trial they contributed to a workshop of key stakeholders, which was held to discuss interpretation and dissemination of the findings. They also provided useful feedback on the final report of the programme, and in particular the lay summary. We have thanked all participants for their involvement and given them details of the website where all published results will be made publically available (www.bristol.ac.uk/healthlines/).

## Results

Participants were recruited between 3 December 2012 and 23 July 2013. Of 7582 people sent information about the study, 1205 (16%) expressed interest and were assessed. Of these, 641 were eligible and randomly allocated to the Healthlines intervention (n=325) or usual care (n=316) arms (fig 1). In total, 597 (93%) of the participants provided follow-up data after six months’ follow-up and 586 (91%) after 12 months’ follow-up (the primary outcome).

**Figure f1:**
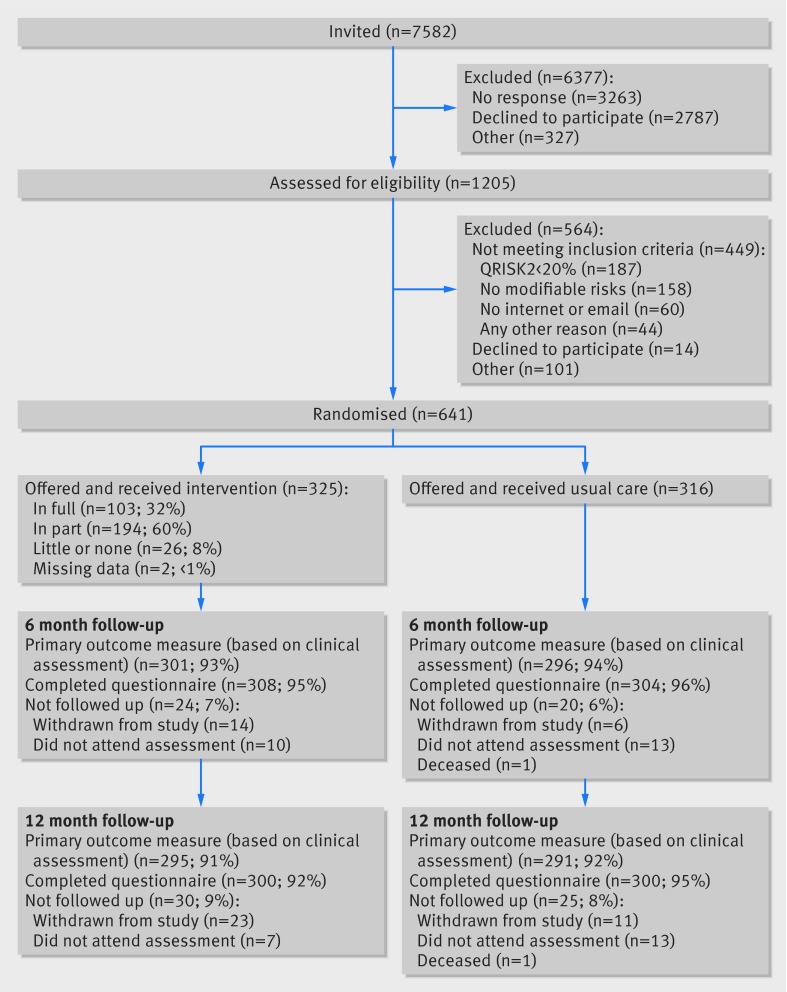
**Fig 1** Flow of participants through trial comparing Healthlines cardiovascular disease intervention with usual care

Table 1 shows the characteristics of participants in the trial. Overall, the participants were at high risk of a cardiovascular event (mean 10 year risk 31%) owing to combinations of modifiable and non-modifiable risk factors. The participants were predominantly white men aged more than 60, and at baseline 356 (56%) were obese (body mass index ≥30), 450 (70%) had a blood pressure of 140 mm Hg or more, and 528 (18%) were current smokers. The two trial arms were well balanced except that there were fewer smokers and more participants with diabetes in the intervention arm. These factors both contribute to the baseline QRISK2 score, which was included as a covariate in all analyses, so we did not conduct additional statistical adjustment for these imbalances.

**Table 1 tbl1:** Baseline characteristics of participants allocated to usual care or Healthlines intervention. Values are percentages (numbers) unless otherwise stated

Characteristics	Usual care (n=316)	Intervention (n=325)
Mean (SD) age at CVD assessment (years)	67.3 (4.7)	67.5 (4.9)
Women	21 (66)	18 (60)
White ethnicity	99 (313)	99 (321)
Current employment situation:	n=311	n=316
Full time	13 (39)	17 (54)
Part time	14 (43)	9 (29)
Unemployed	1 (4)	1 (2)
Unable to work: long term illness/disability	2 (7)	1 (3)
Unable to work: carer	1 (3)	1 (2)
Retired	63 (196)	66 (210)
Homemaker	1 (3)	1 (4)
Other	5 (16)	4 (12)
Occupation (most recent or current):	n=294	n=294
Administrative or secretarial	11 (31)	10 (29)
Associate professional or technical	15 (45)	12 (35)
Elementary*	10 (28)	5 (16)
Managers or senior officials	19 (55)	22 (65)
Personal services†	2 (5)	3 (9)
Process, plant, and machine operatives	5 (15)	6 (17)
Professionals	19 (57)	22 (64)
Sales and customer services	4 (11)	4 (13)
Skilled trades	16 (47)	16 (46)
Highest education qualification:	n=307	n=318
Degree or higher degree	21 (65)	23 (72)
A levels or equivalent	19 (58)	17 (53)
GCSEs/O levels or equivalent	45 (137)	43 (136)
No qualifications	15 (47)	18 (57)
Accommodation:	n=315	n=323
Own accommodation or buying with mortgage	84 (264)	87 (281)
Part rented or rented	15 (46)	12 (40)
Live rent-free	2 (5)	1 (2)
Mean (SD) index of multiple deprivation	16.7 (12.6)	15.5 (11.3)
Clinical data:		
Mean (SD) QRISK2 score	30.8 (9.5)	31.1 (10.2)
Mean (SD) systolic blood pressure (mm Hg)	148.1 (17.6)	147.6 (16.2)
Mean (SD) diastolic blood pressure (mm Hg)	80.0 (10.4)	81.2 (9.6)
Mean weight (SD)	91.9 (18.9)	93.2 (17.3)
Mean (SD) body mass index	30.9 (5.7)	31.2 (5.4)
Mean (SD) total cholesterol level (mmol/L)	4.9 (1.2); n=315	4.9 (1.2);n=324
Mean (SD) total cholesterol:HDL ratio	4.2 (1.4); n=315	4.2 (1.5); n=323
Smoking status:		
Non-smoker	33 (103)	35 (114)
Former smoker	47 (148)	50 (163)
Light smoker	9 (30)	8 (25)
Moderate smoker	5 (17)	5 (16)
Heavy smoker	6 (18)	2 (7)
Taking antihypertensive	61 (193)	64 (209)
Taking lipid lowering drug	49 (153/312)	49 (158/322)
Diabetes	20 (62)	24 (77)
Chronic kidney disease	11 (34)	6 (20)
Atrial fibrillation	6 (20)	7 (23)
Rheumatoid arthritis	3 (8)	2 (6)

CVD=cardiovascular disease; GCSE=general certificate of secondary education; HDL=high density lipoprotein cholesterol.

*Jobs not needing qualifications, such as cleaners.

†For example, care worker, teaching assistant.

### Primary outcome

After 12 months a slightly higher proportion of participants in the intervention arm had improved or maintained their cardiovascular risk compared with those in the usual care arm (50% *v* 42%, respectively; number to treat=13), although this apparent difference had wide confidence intervals and could be due to chance (adjusted odds ratio 1.3, 95% confidence interval 1.0 to 1.9; P=0.08). This conclusion was largely unchanged in our sensitivity analyses (table 2). There was no evidence that the intervention was differentially effective for any of the prespecified subgroups defined by baseline characteristics, although the study was not powered to detect these interaction effects (table 3).

**Table 2 tbl2:** Improving or maintaining cardiovascular risk as a binary outcome. Values are percentages (No/total No) unless stated otherwise

Primary outcome	Usual care*	Intervention†	Adjusted odds ratio (95% CI)	P value
Primary analysis:				
Improved/maintained QRISK2 after 12 months	43 (124/291)	50 (148/295)	1.3 (1.0 to 1.9)	0.08
Secondary analysis:				
Improved/maintained QRISK2 after 6 months	46 (137/296)	48 (145/301)	1.1 (0.8 to 1.5)	0.65
Sensitivity analyses of improved/maintained QRISK2 after 12 months:				
Assuming all participants were one year older	45 (130/291)	52 (153/295)	1.3 (1.0 to 1.9)	0.01
Simple imputation, assuming missing binary outcome is non-response	40 (124/316)	46 (148/325)	1.3 (0.9 to 1.8)	0.11
Multiple imputation	44 (139/316)	50 (163/325)	1.3 (0.9 to 1.8)	0.11
Not including general practice as random effect	43 (124/291)	50 (148/295)	1.3 (1.0 to 1.9)	0.08
Adjusted by days since randomisation to primary outcome assessment	43 (124/291)	50 (148/295)	1.3 (1.0 to 1.9)	0.09

All analyses adjusted by site (Bristol, Sheffield, or Southampton) and baseline QRISK2 score. Analyses are further adjusted by other covariates if specified. General practice included as random effect unless specified otherwise.

*n=296 at six months; n=291 at 12 months; n=316 for imputed data.

†n=301 at six months; n=295 at 12 months; n=325 for imputed data.

**Table 3 tbl3:** Subgroup analyses of primary outcome

**Subgroups**	Improving or maintaining QRISK2 at 12 month follow-up		Adjusted odds ratio* (95% CI)	Interaction P value
	Usual care (n=291)	Intervention (n=295)		
Baseline CVD assessment age group:				
40-59	54 (7/13)	61 (11/18)	1.5 (0.3 to 6.6)	
60-69	44 (78/177)	49 (75/152)	1.2 (0.8 to 1.9)	
≥70	39 (39/101)	50 (62/125)	1.6 (0.9 to 2.8)	0.71
Men	46 (105/227)	51 (125/243)	1.2 (0.9 to 1.8)	
Women	30 (19/64)	44 (23/52)	1.8 (0.8 to 4.0)	0.37
Baseline QRISK2 score:				
17.3-24.9	37 (37/101)	45 (44/98)	1.4 (0.8 to 2.5)	
25.0-29.9	38 (26/68)	44 (35/79)	1.2 (0.6 to 2.4)	
≥30.0	50 (61/122)	58 (69/118)	1.4 (0.8 to 2.4)	0.95
Baseline modifiable risk factor:				
Systolic blood pressure <140 mm Hg	33 (30/90)	41 (35/85)	1.5 (0.8 to 2.8)	
Systolic blood pressure ≥140 mm Hg	47 (94/201)	54 (113/210)	1.3 (0.9 to 1.9)	0.73
Body mass index <30.0	50 (65/131)	52 (67/129)	1.1 (0.6 to 1.8)	
Body mass index ≥30.0	37 (59/160)	49 (81/166)	1.7 (1.1 to 2.6)	0.20
Current smoker	51 (29/57)	53 (23/43)	1.1 (0.5 to 2.5)	
Not current smoker	41 (95/234)	50 (125/252)	1.4 (1.0 to 2.1)	0.55

All analyses adjusted by site (Bristol, Sheffield, or Southampton), baseline outcome, and baseline QRISK2 score. General practice included as random effect.

*Odds ratio comparing intervention with usual care.

### Secondary outcomes

Evidence was lacking of any between group difference in the proportion of participants who improved or maintained their cardiovascular risk after six months’ follow-up (table 2). There was also no evidence of a difference between groups in QRISK2 score when treated as a continuous measure (table 4). However, the intervention was associated with improvements in some of the individual modifiable risk factors that contribute to cardiovascular risk, including reductions in systolic and diastolic blood pressure and weight and body mass index after 12 months’ follow up (table 4). The intervention did not lead to improvements in cholesterol levels (table 4) or smoking rates (table 5).

**Table 4 tbl4:** Secondary outcomes: QRISK2 score as a continuous outcome and individual modifiable cardiovascular risk factors of blood pressure, cholesterol level, weight, and body mass index

**Secondary outcome**	Usual care*		Intervention†		Adjusted difference in means (95% CI)	P value
	Unadjusted mean (SD)	No	Unadjusted mean (SD)	No		
QRISK2 score as continuous variable:						
6 months	31.0 (9.5)	296	31.4 (10.3)	301	0.1 (−0.2 to 0.4)	0.49
12 months	31.2 (10.3)	291	31.3 (10.7)	295	−0.4 (−1.2 to 0.3)	0.27
Systolic blood pressure (mm Hg):						
6 months	141.4 (15.4)	296	141.0 (15.1)	301	0.0 (−1.9 to 1.9)	0.10
12 months	142.2 (16.1)	291	139.6 (14.0)	295	−2.7 (−4.7 to −0.6)	0.01
Diastolic blood pressure (mm Hg):						
6 months	78.0 (9.7)	296	78.2 (9.9)	301	−0.6 (−1.8 to 0.6)	0.34
12 months	78.7 (9.9)	291	76.6 (9.2)	295	−2.8 (−4.0 to −1.6)	<0.001
Total cholesterol level (mmol/L)‡:						
12 months	4.7 (1.1)	288	4.6 (1.2)	295	−0.1 (−0.2 to 0.0)	0.17
Total cholesterol:HDL ratio:						
12 months	4.0 (1.5)	287	4.0 (1.7)	294	−0.1 (−0.2 to 0.1)	0.45
Weight (kg):						
6 months	91.1 (18.4)	296	91.7 (17.7)	301	−0.9 (−1.5 to −0.2)	0.006
12 months	91.2 (19.1)	291	91.3 (17.5)	293	−1.0 (−1.8 to −0.3)	0.008
Body mass index (kg/m^2^):						
6 months	30.6 (5.4)	296	30.7 (5.5)	301	−0.3 (−0.5 to −0.1)	0.006
12 months	30.8 (5.7)	291	30.5 (5.4)	293	−0.4 (−0.6 to −0.1)	0.008

HDL=high density lipoprotein cholesterol.

All analyses adjusted by site (Bristol, Sheffield, or Southampton), baseline QRISK2 score, and baseline outcome. General practice included as random effect.

*n=296 at six months; n=291 at 12 months.

†n=301 at six months; n=295 at 12 months.

‡Cholesterol was not remeasured after six months. Baseline cholesterol measurement was used to calculate QRISK2 at six months.

**Table 5 tbl5:** Secondary outcome: smoking. Values are percentages (numbers) unless stated otherwise

Smoker status	Usual care*	Intervention†	Adjusted odds ratio (95% CI)	P value
Smoker at 6 months:				
Yes	18 (52/296)	15 (45/301)		
No	82 (244/296)	85 (256/301)	0.3 (0.1 to 1.2)	0.01
Smoker at 12 months:				
Yes	18 (52/291)	17 (49/295)		
No	82 (239/291)	83 (246/295)	0.4 (0.2 to 1.0)	0.06

All analyses adjusted by site (Bristol, Sheffield, or Southampton), baseline QRISK2 score, and baseline smoking category (non-smoker, former smoker, light smoker, moderate smoker, heavy smoker). General practice included as random effect.

*n=296 at six months; n=291 at 12 months.

†n=301 at six months; n=295 at 12 months.

Table 6 shows that the intervention was associated with improvements in several of the secondary outcomes. Participants in the intervention arm reported that they improved their diets and increased their level of exercise. They were more likely to adhere to their treatment with statins and antihypertensive drugs. Participants in the intervention arm reported improved access to care and expressed greater satisfaction with the amount of support they received and their overall treatment. They also reported that their care was better organised and coordinated. For ease of presentation, table 6 only shows data on secondary outcomes after 12 months’ follow-up. Findings after six months are available from the authors.

**Table 6 tbl6:** Secondary outcomes at 12 month follow-up

**Secondary outcome**	Usual care (n=300)		Intervention (n=300)		Adjusted difference in means (95% CI)	P value
	Unadjusted mean (SD)	No	Unadjusted mean (SD)	No		
Quality of life (EQ-5D-5L)^34^	0.78 (0.2)	297	0.81 (0.2)	295	0.01 (−0.01 to 0.03)	0.41
Patient behaviours:						
Exercise behaviour (heiQ subscale: health directed behaviour’)*^44^	2.9 (0.8)	294	3.0 (0.8)	297	0.1 (0.0 to 0.2)	0.003
Diet (starting the conversation questionnaire)*^45^	10.3 (2.1)	299	10.9 (2.1)	300	0.6 (0.4 to 0.9)	<0.001
Patient experience:						
Satisfaction with treatment*†	3.7 (0.8)	215	3.9 (0.7)	244	0.1 (0.0 to 0.3)	0.03
Satisfaction with amount of support received*†	2.8 (0.6)	207	3.1 (0.5)	260	0.3 (0.2 to 0.4)	<0.001
Perceived access to care*†	5.5 (1.7)	293	5.8 (1.3)	287	0.3 (0.0 to 0.5)	0.02
Self management skills and self efficacy (heiQ):^44^						
Self monitoring and insight*	3.2 (0.4)	295	3.3 (0.4)	295	0.1 (0.0 to 0.1)	0.07
Constructive attitudes and approaches*	3.3 (0.5)	296	3.4 (0.5)	295	0.0 (0.0 to 0.1)	0.63
Skill and technique acquisition*	3.1 (0.5)	297	3.2 (0.5)	295	0.1 (0.1 to 0.2)	<0.001
Health services navigation*	3.1 (0.6)	296	3.2 (0.5)	297	0.0 (0.0 to 0.1)	0.27
Drug adherence (Morisky)*:**46**						
Anti-hypertensives‡	3.8 (0.5)	194	3.9 (0.3)	203	0.1 (0.0 to 0.2)	0.01
Statins‡	3.6 (0.8)	165	3.8 (0.5)	169	0.2 (0.1 to 0.3)	0.005
Health literacy (eHEALs)*^47^	3.9 (0.7)	296	4.0 (0.7)	295	0.1 (0.0 to 0.2)	0.13
Care coordination (Haggerty):**48**						
Role clarity and coordination*	2.9 (0.5)	247	3.0 (0.3)	263	0.1 (0.0 to 0.1)	0.02
Evidence of care plan*	3.8 (2.1)	209	4.9 (2.0)	236	1.2 (0.8 to 1.5)	<0.001
Overall experience of organisation of healthcare*	3.6 (0.9)	296	3.8 (0.7)	296	0.1 (0.0 to 0.2)	0.04
Self organisation of healthcare*	3.9 (1.1)	283	3.8 (1.0)	287	−0.1 (−0.2 to 0.1)	0.37
Use of telehealth*†‡:						
Online searching	1.6 (0.7)	297	1.6 (0.7)	296	0.1 (−0.0 to 0.2)	0.10
Online forum or group	1.1 (0.3)	295	1.1 (0.4)	298	0.0 (−0.0 to 0.1)	0.29

All analyses adjusted by site (Bristol, Sheffield, or Southampton), baseline outcome (if measured), and baseline QRISK2 score. General practice included as random effect.

*Higher score is more positive (less access difficulties, greater satisfaction).

†Based on scales generated before main trial analysis using principal components analysis and incorporating questions taken from existing validated questionnaires or constructed for this research.

‡Only applicable to those taking antihypertensives or statins.

§Five level ordered categorical variable (never/almost never to daily/almost daily).

After 12 months the incremental cost effectiveness ratio was estimated to be £10 859 ($15 600; €13 800) in 2012/13 prices (incremental cost £138, 95% confidence interval £66 to £211; incremental gain in quality adjusted life years 0.012, 95% confidence interval −0.001 to 0.026). The net monetary benefit at a cost effectiveness threshold of £20 000 was estimated to be £116 (−£58 to £291). The intervention was likely to be cost effective at this threshold after 12 months, with a probability of 0.77. The cohort simulation study showed that the lifetime cost effectiveness of the intervention increased the greater the duration of effect of the intervention on cardiovascular disease risk beyond the follow-up period of the trial. Further details will be published elsewhere.

### Engagement with the intervention

Participants in the intervention arm received a median of 10 (interquartile range 8-12) encounters with the Healthlines service out of a possible maximum of 13 encounters. The mean duration of each encounter was 18 (SD 9.5) minutes. Using a complier average causal effect analysis, we explored whether the number of encounters received in the intervention arm was associated with the primary outcome. The results suggest an increase in effect of the intervention among participants who received all or most of the planned number of encounters (table 7).

**Table 7 tbl7:** Complier average causal effect analysis of primary outcome. Values are unadjusted odds ratios (95% confidence intervals) unless stated otherwise

Amount of intervention received (No of encounters)	Maintenance/reduction in QRISK2 at 12 month follow-up			Partial compliers classified as non-compliers	Partial compliers classified as full compliers
	Usual care (n=291)	Intervention (n=293)		Intervention *v* usual care	Intervention *v* usual care
None (0-2)	43 (124/291)	29 (4/14)			
Partial (3-11)		44 (77/177)			
Full (12-13)		65 (66/102)		2.4 (1.4 to 4.3)	1.4 (1.0 to 1.9)

Three participants who never received Healthlines intervention and two participants who only received unscheduled non-encounter calls are categorised as receiving none of the intervention. Two intervention arm participants had missing data on encounters.

Participants in the intervention arm logged in to the Healthlines website on a median of 14 (interquartile range 3-47) occasions, more than once a month. Overall, 296 (91%) of the participants were given a blood pressure monitor, of whom 200 entered at least one reading, uploading a median of 70 (48-102) blood pressure readings.

Healthlines advisors sent a median of 5 (2-9) letters by email to participants’ doctors. Of these, 138/310 of the participants’ doctors were sent letters advising commencement or review of blood pressure drugs, 32 (10%) were asked to consider statin treatment, 7 (2%) were asked to prescribe orlistat for obesity, and 3 (1%) were asked to prescribe drugs to aid smoking cessation. However, based on data from the medical records, the intervention and control groups did not differ in the number of changes in drugs (starting new treatments or changing dose) for hypertension or lipid lowering, with a median of 0 (0-1) changes for both types of treatment. Similarly, there was no evidence of a difference between the arms in the proportion of participants who reported taking statins or drugs for hypertension, the proportion who had a change in treatment prescribed, or the types of drug prescribed (table 8). These data were not specified as outcomes, but we have presented them to explore the mechanism of effect of the intervention.

**Table 8 tbl8:** Treatment optimisation: cardiovascular risk related drug prescriptions over trial. Values are percentages (numbers) unless stated otherwise

Measure of treatment optimisation	Usual care (n=316)	Intervention (n=325)	Adjusted odds ratio (95% CI): intervention *v* usual care	P value
Experienced at least one change in drugs over 12 month period*:				
Antihypertensive	32 (100)	38 (123)	1.3† (0.9 to 1.8)	0.12
Cholesterol drugs, including statins	22 (71)	26 (84)	1.2† (0.8 to 1.7)	0.33
Self reported use of drugs over 12 month period‡:				
Antihypertensive	68 (196/289)	70 (202/287)		
Statin	57 (165/297)	57 (166/290)		
Prescribed at least one drug over trial period*:				
Antiplatelet	18 (57)	19 (62)		
Cholesterol drugs, including statins	61 (192)	62 (201)		
Smoking cessation	1 (3)	2 (5)		
Obesity drugs	1 (2)	1 (4)		
Antihypertensive	70 (222)	73 (236)		
Prescribed antihypertensive by drug class over trial period*:				
ACE inhibitors or ARBs	50 (159)	52 (170)		
β blockers	18 (58)	16 (52)		
Calcium channel blockers	36 (114)	40 (129)		
Diuretics	29 (90)	29 (93)		
Other	8 (26)	8 (26)		

ACE=angiotensin converting enzyme; ARBs=angiotensin receptor blockers.

*Medical records data.

†Only these between treatment group comparisons are analysed because treatment optimisation was a key aspect of the intervention. Analyses are adjusted by site (Bristol, Sheffield, or Southampton) and baseline QRISK2 score. General practice included as random effect.

‡Questionnaire data.

Over the 12 month period, there was no evidence of a difference between the intervention and control arms in the number of times participants consulted in primary care (mean 11.28 (SD 8.8, n=313) and 11.42 (SD=7.9 n=325), respectively (adjusted incidence ratio 0.99, 0.89 to 1.09, P=0.80).

### Patient safety

Over the course of the trial, 76 adverse events were reported by participants, 38 in each trial arm. Twenty four serious and unexpected events occurred in the usual care arm and 22 in the intervention arm (see supplementary appendix 2). Only one serious event in the intervention arm was likely to be related: a participant was admitted to hospital with low blood pressure, which could have been due to antihypertensive drugs not being reduced after weight loss.

## Discussion

This study suggests a modest benefit from the Healthlines service in terms of the proportion of people reducing or maintaining their cardiovascular disease risk over 12 months. Despite the large sample size, the estimate of effect had wide confidence intervals and could be consistent with no effect or a 90% increase in the odds of reducing or maintaining risk. The results for the primary outcome were not statistically significant either in the complete case analysis or after multiple imputation of missing data. Furthermore, there was no evidence of a difference between the trial arms in average risk, treating QRISK2 as a continuous measure (a secondary outcome). Cardiovascular risk is a composite measure, based on several underlying risk factors. The Healthlines intervention was associated with small but meaningful improvements in several of these factors, including reductions in blood pressure and weight but not in cholesterol level or smoking. It was also associated with improvements in self management behaviours such as diet and physical activity, better adherence to drugs, and greater participant satisfaction with support, access to care, and treatment received. It is important to note that these improvements in self management behaviours would reduce cardiovascular risk beyond the benefit captured in the QRISK2 score and are also likely to reduce risk for many other common and serious diseases, so our focus on cardiovascular risk measured using QRISK2 is likely to be conservative in terms of estimating overall benefit.

The intervention was not successful at promoting optimisation of drug treatment in line with current guidelines, which was a key intended mechanism for reducing blood pressure and cholesterol levels. This is consistent with previous research highlighting the problem of clinical inertia—that treatment is not necessarily intensified in people who fail to reach treatment targets even when regular monitoring shows inadequate control.^35^ Although the observed reduction in cardiovascular risk was small (and could be due to chance), the likely reduction in cardiovascular events in the longer term means that the Healthlines service was likely to be cost effective.

### Strengths and limitations of this study

This is a large and pragmatic trial of a telehealth intervention to reduce cardiovascular risk. It is a complex intervention combining a range of telehealth approaches and has a strong theoretical foundation based on the underlying telehealth in chronic disease (TECH) conceptual model.^18^ The large sample size and high level of participant retention enhance internal validity, whereas the multicentre recruitment and broad inclusion criteria enhance external validity.

The Healthlines intervention incorporates the use of several telehealth approaches, which have reasonable evidence of effectiveness, such as home blood pressure monitoring, and we sought to implement them on a wide scale. Most research studies of telehealth interventions relate to specific technological innovations and can be characterised as efficacy trials, in that they demonstrate the effect of a well defined intervention in people with tightly defined inclusion and exclusion criteria, and who are motivated to use the particular application. These studies may lead to estimates of effect that are exaggerated when compared with the effects observed with wider implementation of the application. By contrast, this trial was pragmatic, testing an intervention as delivered by a mainstream NHS provider in a way that could be rolled out quickly on a wide scale.

This study has several limitations. Firstly, only 16% of those sent information about the study expressed an interest in it. This response rate is not unusual in primary care based trials in which people who may not have an expressed health need are invited to take part in research. Indeed the response rate in this trial was higher than in several other influential trials of related interventions.^36^ ^37^ ^38^ However, if non-respondents differ from respondents because of disinterest in research this could reduce the generalisability of the trial findings. Based on information from 2741 people who gave a reason for non-participation, the most common reasons were related to technology rather than to research: 1491 (54%) had no internet access and 1225 (45%) did not feel confident using computers (people could provide more than one reason).^39^ Many people (n=1135, 41%) did not feel they needed additional support with health problems. It is important to note that less than half of those invited for an NHS Health Check actually attend, and not everyone who smokes or is overweight is motivated to change. We also recognise that telehealth interventions are not necessarily of interest to everyone, and take-up in routine service use may be low. However, healthcare is likely to be increasingly personalised, with different forms of care being chosen by different groups in the population. Telehealth interventions may be useful for a minority of potential participants if (as in the case of increased cardiovascular risk) the total number of people at risk is large.

Secondly, the closure of NHS Direct towards the end of the trial meant that delivery of the intervention was disrupted and many participants received less than the full course of intervention encounters. However, that we were able to move the service quickly to another provider demonstrates the transferability of the approach. Thirdly, we analysed a large number of secondary outcomes in order to capture the range of potential effects from this complex intervention, but this raises the possibility of some apparent differences being due to chance because of multiple testing. Fourthly, the sample size was chosen pragmatically and assumed that 35% of participants in the control arm would maintain or reduce their cardiovascular risk over 12 months. In the trial, a higher than anticipated proportion of those in the control group achieved this, perhaps because of the impact of the NHS Health Checks programme.^26^ This reduced the power of the study to detect differences between the intervention and control groups, but this will have been mitigated to some extent by the fact that we recruited and successfully followed up more patients than anticipated. Fifthly, the study was limited to patients aged less than 75 years (because this is the age range in which QRISK2 has been validated and is also the age group targeted by NHS Health Checks), but this intervention could potentially also help older people. The study also excluded people without access to the internet; however, the proportion of the population with access is increasing rapidly.

Finally, the use of cardiovascular risk as a composite outcome has limitations because the QRISK2 score is strongly dominated by non-modifiable factors such as age and sex. We chose to analyse the QRISK2 as a binary measure of “response to treatment” for the primary outcome because this approach is sensitive to changes in modifiable risk factors. The number of patients needed to treat to gain benefit from the intervention was 13. However, because only a minority of participants benefited, there was no statistically significant change in QRISK2 averaged across all participants when analysed as a continuous variable (a secondary outcome). Nevertheless, the small changes in modifiable risk factors observed in this trial are likely to be associated with meaningful benefits. Based on the systematic review by Law et al,^40^ the reductions in blood pressure observed in this trial would lead to a 23% reduction in the relative risk of stroke and a 15% reduction in the relative risk of a heart attack. The combined effect (along with the reduction in weight) suggests that these small changes in modifiable risk factors are likely to be worthwhile, particularly at a population level when applied to the large number of people at high risk of cardiovascular disease.

### Comparison with other studies

This was a trial of the implementation of the combined use of a range of telehealth interventions to deal with cardiovascular risk factors. The results are broadly consistent with earlier trials, which have studied different components of the intervention in isolation to reduce individual risk factors. A systematic review of trials of blood pressure self monitoring showed that this was associated with small reductions in both systolic and diastolic blood pressure of a similar size to those achieved in the Healthlines cardiovascular disease risk trial.^23^ A Cochrane review found that computer based interactive interventions for weight loss were associated with a mean weight loss of 1.5 kg (95% confidence interval 0.9 to 2.1 kg) compared with no or minimal intervention, an effect which is also consistent with our findings.^25^ Systematic reviews on internet based telehealth interventions for smoking cessation show mixed effects, although mobile phone based interventions are effective and telephone quitlines can improve cessation rates in those people who proactively contact them.^24^ ^41^ ^42^ It is important to note that the above reviews were all based on people who had the risk factor of interest, and many trials only included those who were motivated to change the specific risk factor. In the Healthlines cardiovascular disease risk trial only a proportion of participants had raised blood pressure, were obese, or were smokers at baseline, and they were not necessarily motivated to change the main factor contributing to their risk, so effects are likely to be smaller than in studies on specific risk factors.

The Healthlines intervention tested in this trial had a similar impact on blood pressure reduction as the earlier trials by Bosworth et al, which used a similar behavioural management system (but provided by nurses rather than lay staff and without incorporating the use of internet resources).^28^ ^29^ However, the Healthlines trial had less impact than two trials from the United States, which involved blood pressure self management with pharmacist management of drugs by phone or over the internet.^36^ ^37^ The involvement of pharmacists to directly alter drugs without the intermediate step of sending advice to primary care doctors may be associated with more effective optimisation of treatment but could be problematic in a routine primary care context, when patients often have comorbidities and other factors need to be considered in treatment decisions.

Two systematic reviews of telehealth interventions to reduce overall cardiovascular risk have recently been published.^14^ ^43^ Several studies demonstrated small improvements in blood pressure and weight, findings for cholesterol were equivocal, and there was no evidence of increased rates of smoking cessation. Our results are consistent with these findings but provide much stronger evidence from a large, rigorous and pragmatic trial.

### Implications for clinicians and for policy

The development of the Healthlines service reflected a conceptual framework that was based on promoting self management, improving drug adherence and optimisation of drug treatment, coordination of care, and the active engagement of patients and primary care clinicians.^18^ This randomised controlled trial shows modest but cost effective benefit in cardiovascular risk reduction. Delineating how components of a multifaceted intervention work, alone or in combination; their effect on doctor practice in terms of optimisation or intensification of medicines and their effect on behaviour modification by patients is complex. What is clear is that patients who engaged with the intervention seem to gain the most in terms of cardiovascular risk, but some components of the intervention, particularly optimisation or intensification of drugs, were ineffective. To improve the effectiveness of the intervention it will be important to target it at those who are motivated to change their risk behaviours, and to improve communication with primary care prescribers about drug treatment recommendations.

### Conclusions

Optimism about the potential of telehealth approaches to improve the accessibility, convenience, and efficiency of healthcare has been considerable. This study adds to the growing evidence base, which suggests that healthcare delivery systems based on telehealth may be associated with some benefits, although these should not be assumed. However, this study has demonstrated the feasibility of delivering an intervention on a wide scale at relatively low cost and using non-clinically trained health advisors supported by computerised algorithms. This increases the capacity of the healthcare system to provide an intervention to large numbers of people. Further development of this type of intervention is justified to increase the effectiveness of the Healthlines service approach.

**What is already known on this topic**

Given the increasing prevalence of long term health conditions, it is necessary to explore new ways to deliver healthcare and to support self management to expand provision of care at low cost
There is considerable optimism among policy makers that greater use of digital health technologies (“telehealth”) in combination with new ways of working could transform healthcare delivery, helping the UK national health service to be sustainable
Evidence about the effectiveness of telehealth interventions is equivocal, with some benefits from specific technologies but little evidence of effectiveness in real world implementation

**What this study adds**

Some evidence suggests that an intervention combining the use of a range of digital health technologies with telephone support from trained lay health advisors, leads to a modest improvement in overall cardiovascular risk for a minority of participants
The intervention had no impact on average cardiovascular risk but was associated with improvements in specific cardiovascular risk factors and health behaviours and patient perceptions of support and access to care

## Web extra

Extra material supplied by authors

**Web appendix:** supplementary information

## Notes

We thank the patients, healthcare professionals, health information advisors, and other NHS Direct staff who contributed time and effort to make this trial possible; Sarah Williams at Solent NHS Trust who took over responsibility for hosting the intervention when NHS Direct closed; Steve Bellerby who managed implementation of the Healthlines software at both trusts; members of the trial steering and data monitoring committees, particularly Michelle McPhail and Anne Jacob who were patient and public representatives; Hayden Bosworth for permission to adapt his behavioural intervention, and both he and Felicia McCann for their help with adaptation; Roberta Ara for input into the design and implementation of the lifetime cost effectiveness cohort simulation model; Glyn Lewis and Simon Brownsell who were applicants on the research programme grant but not directly involved in this trial; the Primary Care Research Network (now, NIHR Clinical Research Network) for assisting us with recruitment of general practitioners; and Frederika Collihole, Richard Campbell, Ben Davies, Lorna Duncan, Diane Beck, and Janet Cooke who supported participant recruitment, data entry, and trial administration. This study was designed and delivered in collaboration with Bristol Randomised Trials Collaboration (BRTC), a UKCRC Registered Clinical Trials Unit in receipt of National Institute for Health Research CTU support funding.

Contributors: CS, AO’C, SH, JN, SL, LY, TF, AR, CP, and AAM developed the protocol for the study, obtained funding, provided methodological advice, and supervised the conduct of the trial. CS led protocol development and the funding application, acted as chief investigator with overall responsibility for the conduct of the trial, and led the drafting of the article. AO’C supervised the conduct of the trial in Sheffield. CT, MSM, and LE acted as trial managers, coordinating the conduct of the trial across the centres. LE, AF, KG, and KH undertook participant recruitment and follow-up, data collection, and data entry. DG developed the statistical analysis plan and undertook the statistical analysis. PD undertook the economic analysis. SL coordinated development and delivery of the intervention with NHS Direct. AAM supervised the statistical analysis. All authors, external and internal, had full access to all of the data (including statistical reports and tables) in the study and can take responsibility for the integrity of the data and the accuracy of the data analysis. All authors approved the final version of this manuscript. CS is guarantor.

Ethical approval: This study was approved by the National Research Ethics Service Committee South West–Frenchay (reference 12/SW/0009). All participants provided informed consent to take part in the trial.

Data sharing: The research team will consider reasonable requests for sharing of patient level data. Requests should be made to CS. Consent for data sharing was not obtained but the presented data are anonymised and risk of identification is low.

Transparency: The lead author (manuscript’s guarantor) affirms that the manuscript is an honest, accurate, and transparent account of the study being reported; that no important aspects of the study have been omitted; and that any discrepancies from the study as planned and registered have been explained.
