# Supplementary material for: Telehealth for patients at high risk of cardiovascular disease: pragmatic randomised controlled trial
Source: BMJ. 2016 Jun 1;353:i2647. doi: 10.1136/bmj.i2647 (PMC4896755; doi:10.1136/bmj.i2647)
Supplement: Supplementary file 1 — Web appendix: supplementary information [file salc031512.ww1_default.pdf]

Appendix 1 Process of conducting health checks for baseline and primary outcome assessment

Health checks for assessment of eligibility and measurement of the primary outcome were conducted by practice nurses or health care assistants who had been trained by the research team. The nurse or health care assistant took four BP readings at one-minute resting intervals using an OMRON M3 upper-arm BP monitor. The first two readings were taken on alternate arms, and the third and fourth readings were taken on whichever arm gave the highest reading. The average systolic and diastolic pressures based on the final two readings were recorded. The participant's BMI was calculated after measuring their height and weight. Smoking status was assessed by patient self-report and validated using a carbon monoxide monitor (COmpact Smokerlyzer, Bedfont Scientific). Further information necessary to calculate cardiovascular risk using the QRISK2 algorithm was collected from patient self-report and the medical records (e.g. ethnicity, age, family history, diagnosis of diabetes etc.). A non-fasting blood sample was taken to measure total and high-density lipoprotein cholesterol levels unless a result was available for this test in the previous three months in which case those results were used instead. The information from the health check was sent to the research team, who used this to calculate the QRISK2 score.

Appendix 2 Adverse events reported throughout the CVD risk trial by relatedness to the intervention and seriousness

	**Usual care**				**Intervention**			
	**Not related**	**Unlikely to be related**	**Possibly related**	**Definitely related**	**Not related**	**Unlikely to be related**	**Possibly related**	**Definitely related**
Not serious	13	0	0	1	8	2	3	2
Serious, unexpected	24	0	0	0	19	2	1	0
Serious, expected	0	0	0	0	0	1	0	0
